# Successful Treatment of a Patient with Giant Cell Vasculitis (Horton Arteritis) with Tocilizumab a Humanized Anti-Interleukin-6 Receptor Antibody

**DOI:** 10.1155/2012/639612

**Published:** 2012-04-03

**Authors:** Alfredomaria Lurati, Luca Bertani, Katia Angela Re, Mariagrazia Marrazza, Daniela Bompane, Magda Scarpellini

**Affiliations:** Rheumatology Unit, Fornaroli Hospital, 22013 Magenta, Italy

## Abstract

Giant cell arteritis (GCA) is the most common form of systemic vasculitis in adults, affecting preferentially medium-large size arteries. Here we report a case of a female with a diagnosis of GCA based on temporal artery biopsy, successfully treated with tocilizumab, a humanized anti-interleukin-6 receptor antibody.

## 1. Introduction

Giant cell arteritis (GCA) is an immune-mediated disease characterised by granulomatous infiltrates in the wall of medium- and large-size arteries including the aorta with vascular damage and formation of stenosis and aneurysms. It affects over-50-year-old individuals with an annual incidence varying between 6 and 27 cases per 100 000 persons worldwide. Glucocorticoids remain the treatment of choice, but relapses may occur when dosages are tapered [1, 2]. Immunosuppressive drugs as methotrexate and azathioprine have been used in controlling disease and tapering steroids. Anti-TNF agents as Etanercept and Infliximab have been used too. Methotrexate demonstrated some efficacy; data about azathioprine and anti-TNF drugs remain however, debatable. Various data about the role of interleukin 6 (IL-6) in pathogenesis of GCA have been published [3, 4]. We report a case of a patient a 54 year-old woman with GCA treated between January 2011 until now with tocilizumab a humanized anti interleukin-6 receptor antagonist.

## 2. Case Presentation

A 67-year-old Caucasian woman with no prior medical history was admitted to our rheumatology unit in September 2009 for acute onset of stiffness and pain in the shoulder and pelvic musculature, headache accompanied by fever and malaise. Initial laboratory findings showed increased C-reactive protein level at 9.5 mg/dL, haemoglobin of 10.2 g/dL. Rheumatoid factor, Waaler-Rose reaction, and anticitrullinated cyclic were negative. Antinuclear autoantibodies and anti-dsDNA as well as cANCA and pANCA were negative. Biopsies of temporal arteries were performed before any treatment, showing lesions of a granulomatous vasculitis with giant cells. A diagnosis of GCA was so performed. Subsequently we performed a whole body ^18^F-FDG-PET/CT that revealed metabolic hyperactivity in ascendant aorta and axillary and subclavian arteries. A contrast-enhanced MR angiography (MRA) of the aorta documented mural thickening and hyperenhancement in contrast enhanced T1- and T2-weighted images, findings characteristic for active vasculitis. From October 2009 the patient received prednisone 0.5 mg/kg/day (25 mg/die) with a prompt clinical and serological response. During the steroid tapering (2.5 mg/week), we observed at a dose of 12.5 mg/day a relapsing of the symptoms and a rising of CRP levels to 3.7 mg/dL.

Informed consent by the patient and approval by the local ethics committee were so obtained for the injection of tocilizumab 8 mg/kg every 4 weeks as steroid-sparing agent. First dose was administered in January 2011, in combination with prednisone 10 mg/day. After 3 months serum CRP lowered to 0.1 mg/dL, haemoglobin levels raise to 11.5 mg/dL, and pain in the shoulders and pelvic promptly improved. Steroids dose was so tapered until suspension in 1 month. Despite steroid suspension CRP, ESR, and Haemoglobin levels remained until now in normal range. A PET/TC and a MRA were performed in October 2011, revealing significant lowering in metabolic activity of vasculitis in all districts.

## 3. Discussion

Several studies of cytokines in temporal artery biopsy sections of patients with GCA revealed overexpression of IL-6 messenger RNA and protein, indicating that IL-6 may play a role in the development of GCA. Although there have been no reports regarding treatment of GCA with tocilizumab, Nishimoto et al. found that treatment of a patient with Takayasu arteritis with tocilizumab improved the clinical manifestations and abnormal laboratory findings [4–6].

Our case seems to confirm the role of tocilizumab as a treatment option for GCA.
